# Capsule Endoscopy in the Diagnosis of Hookworm‐Induced Acute Intestinal Bleeding—A Case Report

**DOI:** 10.1002/ccr3.72791

**Published:** 2026-05-24

**Authors:** Chukwuka Elendu, Eunice K. Omeludike, Victor B. Bieh, Sobechukwu F. Chiegboka, Olusegun A. Aladesanmi, Ebubechukwu Ibe, Ezinnebuchi P. Uzor‐Lawrence, Emeka E. Ukoha

**Affiliations:** ^1^ Federal University Teaching Hospital Owerri Nigeria; ^2^ Piedmont Athens Regional Medical Centre Athens Georgia USA; ^3^ University of Port Harcourt Port Harcourt Nigeria; ^4^ Saint Michael Medical Center Newark New Jersey USA; ^5^ Afe Babalola University Ado‐Ekiti Nigeria; ^6^ Igbinedion University Okada Nigeria

**Keywords:** capsule endoscopy, hookworm infection, parasitic anemia, small‐bowel hemorrhage, suspected small bowel bleeding

## Abstract

In endemic regions, unexplained small‐bowel bleeding should prompt consideration of parasitic etiologies even when routine investigations are unrevealing. Advanced small‐bowel imaging can directly demonstrate active sources of hemorrhage, enabling timely targeted therapy, preventing unnecessary procedures, and reducing morbidity associated with delayed or missed diagnosis.

## 1 Introduction and Background

Hookworm infection is a significant public health problem in many low‐ and middle‐income countries, affecting an estimated 400–500 million people worldwide, particularly in regions with poor sanitation and limited access to clean water [1]. Although most infections are asymptomatic or present with chronic iron‐deficiency anemia due to low‐grade blood loss, severe gastrointestinal bleeding is rare but potentially life‐threatening [2]. Acute overt intestinal bleeding caused by hookworm infection is uncommon and may present significant diagnostic challenges, particularly when conventional endoscopic evaluations fail to identify a bleeding source [3].

Hookworms, primarily *Ancylostoma duodenale* and *Necator americanus* , attach to the small intestinal mucosa and cause mucosal injury through mechanical trauma, secretion of anticoagulant substances, and repeated migration between attachment sites, all of which can contribute to hemorrhage [4]. However, the small intestine remains a diagnostic blind spot for standard endoscopy, often resulting in delayed or missed diagnoses in patients with suspected small bowel bleeding (SSBB) [5]. Capsule endoscopy has emerged as a minimally invasive and highly sensitive modality for visualizing the entire small bowel, allowing direct identification of mucosal lesions, parasites, and active bleeding sites [6].

In recent years, capsule endoscopy has gained prominence in the evaluation of SSBB, with reported diagnostic yields exceeding 60% in selected patient populations [5, 7]. Although its role in parasitic infections is less frequently emphasized, capsule endoscopy is increasingly recognized as a valuable diagnostic tool in endemic regions where hookworm infection is highly prevalent [6]. In this report, we describe the use of capsule endoscopy to diagnose hookworm‐induced acute intestinal bleeding, highlighting its diagnostic value and reinforcing the importance of considering parasitic etiologies in cases of unexplained gastrointestinal hemorrhage in endemic settings.

## 2 Case Description

The patient was a 42‐year‐old male subsistence farmer residing in a rural community within a known hookworm‐endemic region who presented to the emergency department with a five‐day history of progressive generalized weakness, exertional dizziness, and passage of black, tarry stools, with intermittent dark red blood per rectum. He described the bleeding as painless and denied hematemesis, coffee‐ground vomiting, abdominal pain, bloating, or changes in bowel frequency. There was no history of recent febrile illness, weight loss, night sweats, anorexia, or chronic gastrointestinal symptoms, and he had no prior diagnosis of gastrointestinal, hepatic, or hematologic disease and had never undergone endoscopic evaluation. He denied the use of nonsteroidal anti‐inflammatory drugs, aspirin, anticoagulants, herbal preparations, or alcohol, and there was no family history of bleeding disorders or gastrointestinal malignancy (clinical timeline summarized in Table 1).

**TABLE 1:** Clinical timeline of the patient's diagnostic evaluation and management.

Time point	Clinical events
Day‐5 (Symptom onset)	Progressive weakness, dizziness, and melena with intermittent hematochezia
Day 0 (Hospital presentation)	Severe symptomatic anemia; Hb 5.6 g/dL. FOBT positive; initial stool microscopy negative
Day 0	CT pulmonary angiography revealed **multiple** pulmonary emboli
Day 1	Upper gastrointestinal endoscopy demonstrated **motile** intestinal helminths attached to duodenal mucosa with active bleeding
Day 1–2	Colonoscopy revealed dark blood throughout colon without identifiable source
Within 48 h	Repeat Hb 6.9 g/dL after initial resuscitation and supportive management
Day 2	Capsule endoscopy (PillCam SB3) demonstrated multiple intestinal helminths attached to jejunal mucosa with active bleeding
Day 2	Stool concentration test (formalin–ethyl acetate) demonstrated numerous hookworm ova
Day 2–4	Albendazole 400 mg daily for 3 days initiated with supportive care
6‐month follow‐up	CT pulmonary angiography showed complete resolution of pulmonary emboli and clinical recovery

Upon questioning, the patient reported long‐term occupational exposure to moist soil through barefoot farming and limited access to potable water and sanitation. He denied recent travel or prior hospitalizations and presented to the hospital when he began experiencing shortness of breath with minimal exertion.

On arrival, the patient appeared pale, fatigued, mildly tachypneic, and visibly weak, with vital signs showing a blood pressure of 96/58 mmHg, heart rate of 112 beats per minute, respiratory rate of 22 breaths per minute, oxygen saturation of 97% on room air, and temperature of 36.8°C. He demonstrated marked conjunctival pallor with cold extremities and delayed capillary refill, and cardiovascular examination revealed sinus tachycardia with normal heart sounds and no murmurs, rubs, or gallops. The lungs were clear to auscultation bilaterally, and the abdomen was soft and non‐tender with no guarding, rebound tenderness, or palpable masses; the liver and spleen were not enlarged, and bowel sounds were normal. Digital rectal examination revealed black stool mixed with dark red blood without fissures, hemorrhoids, or rectal masses.

Initial laboratory testing demonstrated severe microcytic anemia, with a hemoglobin level of 5.6 g/dL at presentation, hematocrit of 17.8%, and mean corpuscular volume of 72 fL. Red cell distribution width was elevated, consistent with anisocytosis, with a mildly increased white blood cell count of 12.4 × 10^9^/L and platelet count of 410 × 10^9^/L, while iron studies showed a low serum ferritin of 8 ng/mL and reduced transferrin saturation of 7%, indicating iron deficiency. Renal function tests, including serum urea and creatinine, and liver function tests, including alanine aminotransferase, aspartate aminotransferase, bilirubin, and alkaline phosphatase, were within normal limits. Coagulation parameters, including prothrombin time and activated partial thromboplastin time, and serum electrolyte levels were within normal limits, while fecal occult blood testing was positive, and an initial single stool sample examined using direct saline wet mount microscopy did not reveal ova or parasites.

**FIGURE 1:**
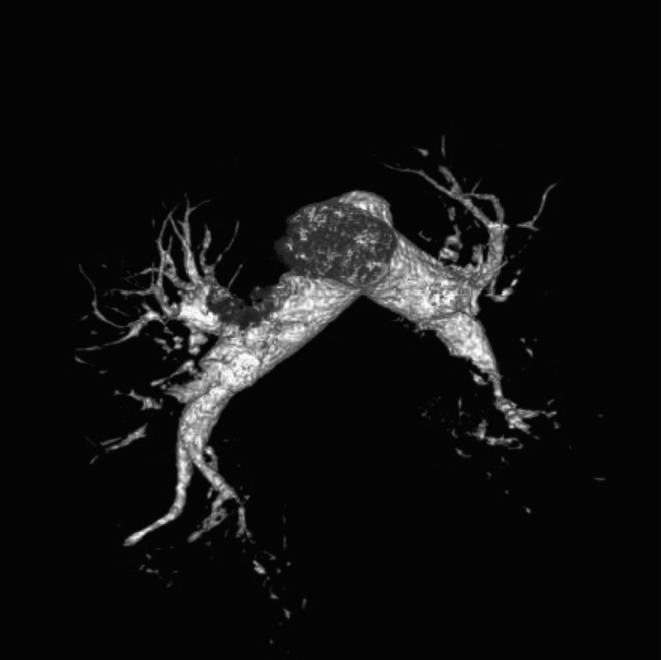
Contrast‐enhanced CT pulmonary angiography demonstrating multiple filling defects within the pulmonary arterial branches consistent with acute pulmonary emboli in the setting of severe gastrointestinal bleeding–associated hypercoagulability.

Given the severity of acute gastrointestinal bleeding and profound anemia, which are recognized to confer a hypercoagulable state, contrast‐enhanced CT angiography was performed to assess for thromboembolic complications and demonstrated multiple pulmonary emboli (Figure 1); this finding prompted careful cardiopulmonary monitoring during the subsequent diagnostic and therapeutic course.

**FIGURE 2:**
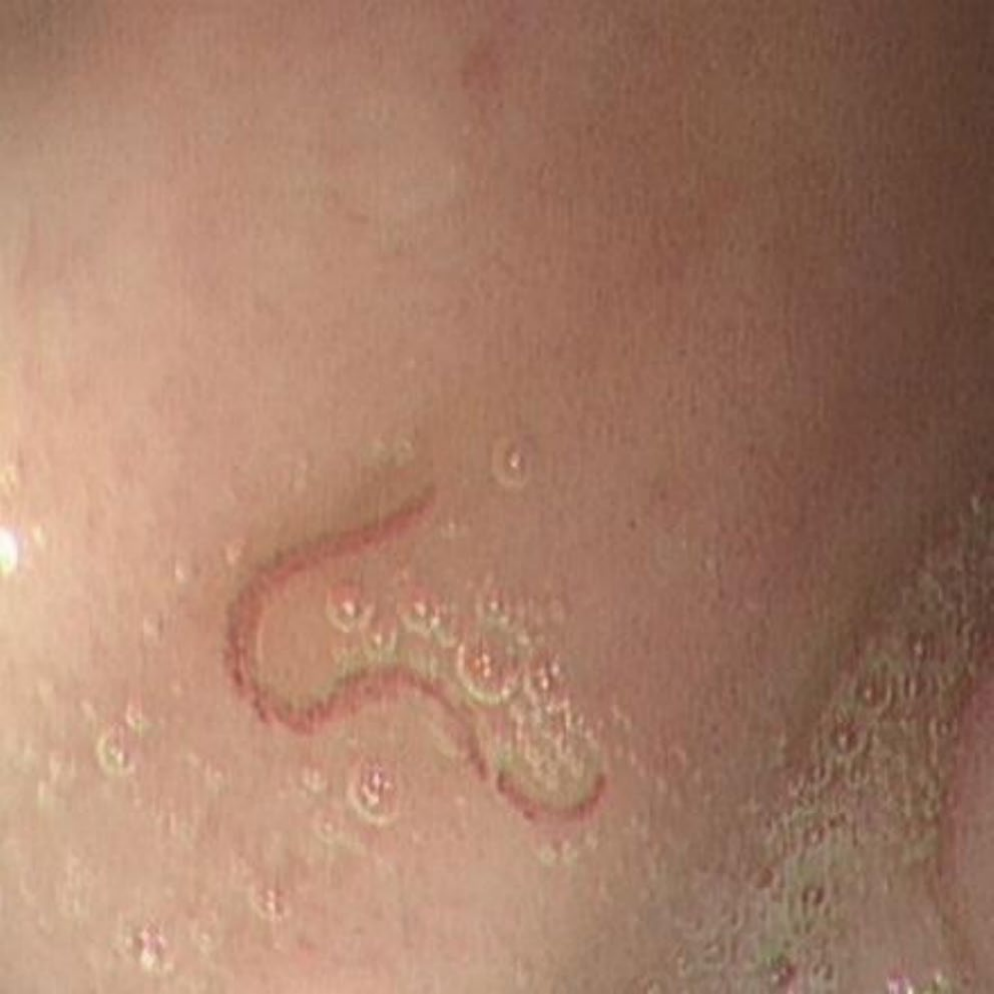
Upper gastrointestinal endoscopy showing a motile intestinal helminth attached to the duodenal mucosa with surrounding erythema and focal bleeding at the site of attachment, consistent with hookworm infection.

The patient was admitted for evaluation of overt gastrointestinal bleeding with severe anemia and continued to pass melena with intermittent maroon‐colored stools. Following initial resuscitation and supportive management, repeat laboratory testing showed a hemoglobin level of 6.9 g/dL within 48 h of admission. Upper gastrointestinal endoscopy performed within the first 24 h demonstrated normal esophageal and gastric mucosa; however, multiple red, motile worms were visualized swimming freely and intermittently attaching to the duodenal mucosa, with surrounding erythema, focal erosions, and areas of slow, active oozing of blood (Figure 2). The organisms were slender and cylindrical with sinuous movements and were clearly distinguishable from food residue or mucus. The underlying mucosa at attachment sites was hyperemic and friable with pinpoint hemorrhages persisting after worm detachment, and no peptic ulcers, vascular ectasias, masses, or varices were observed in the esophagus, stomach, or duodenum, nor were there stigmata of recent nonparasitic bleeding. Although the parasites were clearly visualized during endoscopy, endoscopic retrieval was not attempted because the organisms were highly motile and intermittently detaching from the mucosa, making capture technically challenging during the procedure. Consequently, definitive morphological identification to the species level could not be performed. The visualization of motile worms in the duodenum raised strong suspicion of intestinal helminthiasis as a contributing factor to the patient's bleeding, although the extent of small‐bowel involvement could not be determined solely by conventional endoscopy.

Colonoscopy was subsequently performed following adequate bowel preparation and revealed dark blood throughout the colon but no mucosal lesions, ulcers, polyps, angiodysplasia, or neoplastic changes that could account for the hemorrhage. The terminal ileum was intubated and appeared macroscopically normal without ulceration, nodularity, or visible parasites, suggesting that the bleeding source was proximal to the terminal ileum and beyond the reach of standard endoscopy, making the small intestine the most likely site of ongoing blood loss.

**FIGURE 3:**
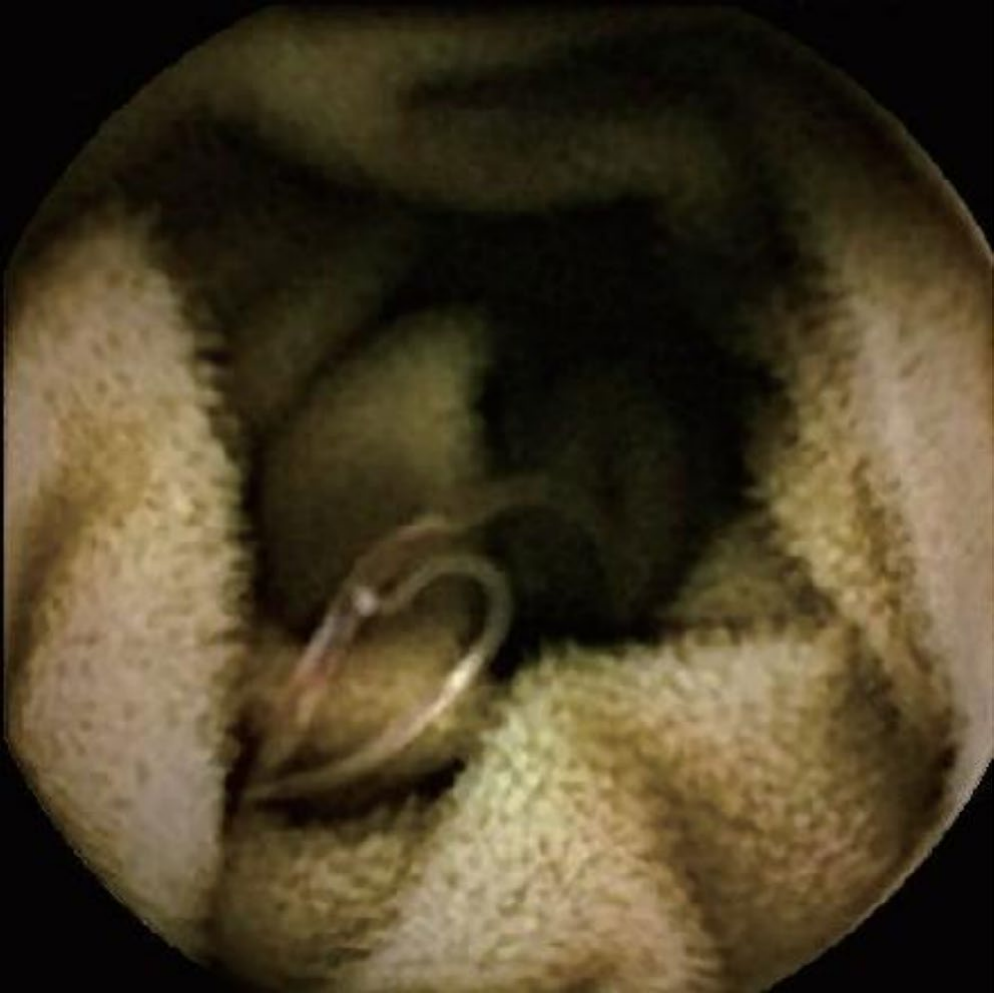
Capsule endoscopy image showing an intestinal helminth attached to the jejunal mucosa with its anterior end embedded in the epithelium and surrounding mucosal erythema, findings consistent with hookworm infection.

Because of persistent overt bleeding with nondiagnostic conventional endoscopic evaluations, capsule endoscopy was arranged to visualize the small bowel, and after standard bowel preparation, the patient swallowed the capsule without difficulty, with adequate image transmission throughout its transit. The examination was performed using a PillCam SB3 capsule endoscopy system (Medtronic, Dublin, Ireland). Review of the capsule endoscopy recording revealed striking abnormalities in the jejunum and proximal ileum, with multiple slender, whitish‐brown, cylindrical, motile organisms attached to the intestinal mucosa over extended segments of bowel (Figure 3), embedded by their anterior ends with posterior portions moving freely in the lumen, and displaying morphology, motility, and attachment patterns consistent with hookworms.

**FIGURE 4:**
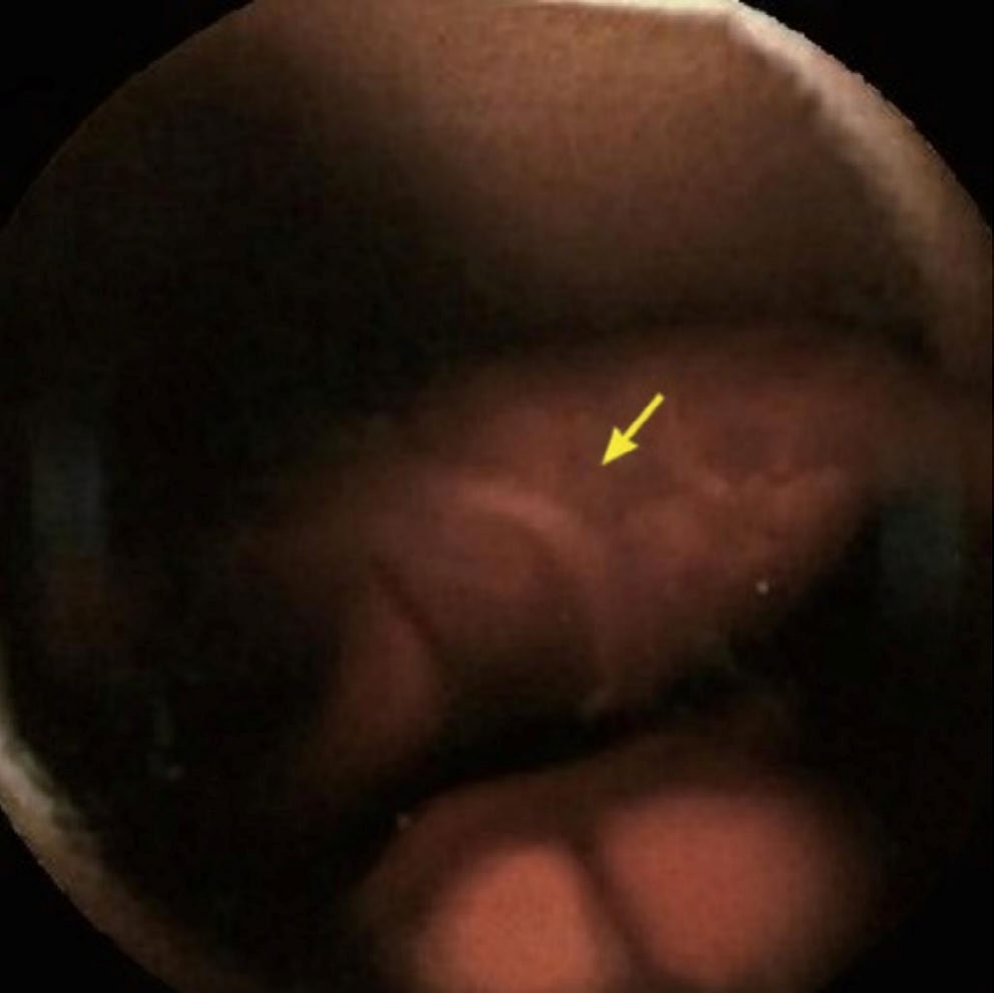
Capsule endoscopy image showing an intestinal helminth embedded in the jejunal mucosa, with the arrow indicating the parasite's attachment site associated with focal hemorrhage and mucosal injury, consistent with hookworm infection.

At numerous sites, the parasites were associated with localized mucosal injury with surrounding erythema, superficial erosions, and small pools of fresh blood, and in several sequential frames, individual hookworms were observed detaching from one site and migrating a short distance before reattaching to nearby mucosa, leaving behind punctate bleeding points and small adherent clots. In one particularly illustrative frame, a hookworm was captured with its anterior end deeply embedded in the mucosa, with an arrow highlighting the attachment point associated with a focal hemorrhagic lesion (Figure 4), providing direct visual confirmation of active feeding and mechanical disruption of the epithelial surface.

The burden of infestation was most pronounced in the proximal and mid‐jejunum, where clusters of worms densely populated limited bowel segments, producing diffuse mucosal injury with widespread erythema, scattered erosions, and continuous oozing of blood into the lumen. In contrast, intervening segments appeared relatively preserved with normal villous architecture and no evidence of inflammatory bowel disease, ischemia, or neoplasia, and no tumors, ulcers, strictures, vascular malformations, capsule retention, or obstruction were identified elsewhere in the small intestine.

The active bleeding associated with the parasites provided a clear anatomical and mechanistic explanation for the patient's severe anemia and ongoing melena. Unlike the typical presentation of hookworm infection with chronic occult blood loss, this patient showed multiple sites of simultaneous hemorrhage consistent with an unusually heavy parasitic burden. Fresh blood was repeatedly visualized pooling in the lumen adjacent to recently vacated attachment sites, with slow trickles emanating directly from the mucosa after worm detachment.

The morphology of the organisms—elongated, slightly curved, with tapered anterior ends and active sinuous movement—was characteristic of hookworms, most likely *Ancylostoma duodenale* or *Necator americanus* . Although species differentiation was not possible based solely on endoscopic appearance, the combination of epidemiologic exposure, visualized parasites, and mucosal hemorrhage was diagnostic of hookworm‐induced intestinal bleeding.

To corroborate the endoscopic findings, repeat stool examination was performed on two additional samples using a formalin–ethyl acetate concentration technique, which demonstrated numerous hookworm ova, confirming active infection and correlating with the extensive parasitic burden observed on capsule endoscopy. The temporal relationship between the patient's overt gastrointestinal bleeding, severe iron‐deficiency anemia, and the direct visualization of feeding hookworms provided compelling evidence of a causal relationship.

No complications occurred during capsule transit, and the device was passed spontaneously. The patient remained hemodynamically stable throughout the diagnostic process despite laboratory evidence of ongoing blood loss until the source was identified.

## 3 Differential Diagnosis, Treatment Plan, and Follow‐Up

**FIGURE 5:**
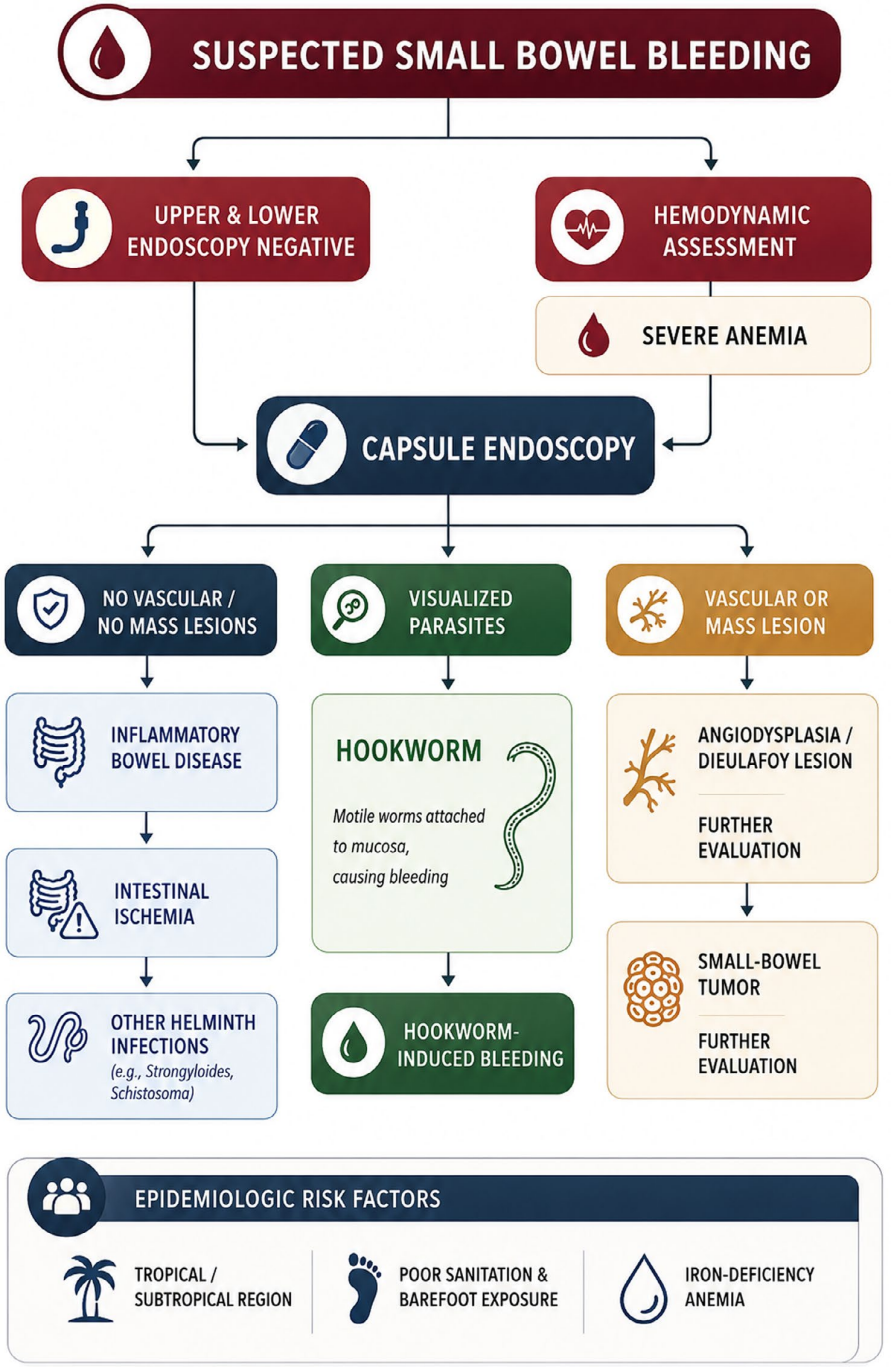
Diagnostic flowchart illustrating the stepwise approach to suspected small bowel bleeding (SSBB) in endemic settings, highlighting capsule endoscopy–based differentiation between parasitic, vascular, and neoplastic small‐bowel causes.

Although the clinical history and endoscopic findings strongly suggested a parasitic cause of gastrointestinal bleeding, a focused differential diagnosis for SSBB was considered (Table 2; Figure 5) [8, 9, 10, 11]. Given the direct visualization of motile worms attached to the intestinal mucosa with active bleeding on capsule endoscopy, these alternative causes were deemed unlikely. Taken together, the patient's epidemiologic exposure, capsule endoscopy findings, and confirmatory stool microscopy established hookworm‐induced intestinal hemorrhage as the definitive diagnosis [1].

**TABLE 2:** Differential diagnosis of suspected small‐bowel bleeding in endemic settings.

Etiology	Typical clinical features	Endoscopic/imaging findings	Reason excluded in this case	Supporting reference
Peptic ulcer disease	Epigastric pain, NSAID use, melena, hematemesis	Gastric or duodenal ulcers with visible vessels or bleeding	Upper endoscopy showed no ulcers, erosions, or bleeding stigmata	Laine and Jensen [8]
Portal hypertensive bleeding	Known liver disease, splenomegaly, ascites, varices	Esophageal or gastric varices, portal gastropathy	No varices or signs of chronic liver disease were present	Laine and Jensen [8]
Small‐bowel angiodysplasia	Painless intermittent bleeding, anemia	Dilated ectatic vessels on capsule endoscopy	Capsule endoscopy showed no vascular malformations	Gerson et al. [9]
Dieulafoy lesion	Sudden massive bleeding, normal surrounding mucosa	Focal arterial bleeding from small mucosal defect	No focal arterial bleeding or exposed vessels seen	Gerson et al. [9]
Small‐bowel tumors (GIST, adenocarcinoma, lymphoma)	Weight loss, anemia, obstruction, bleeding	Mass lesion, mucosal distortion, luminal narrowing	Capsule endoscopy revealed no masses or obstructive features	Dabaja et al. [10]
Inflammatory bowel disease	Chronic diarrhea, abdominal pain, systemic inflammation	Ulcers, mucosal edema, friability	Normal villous architecture without inflammation	Feuerstein and Cheifetz [11]
Intestinal ischemia	Severe abdominal pain, acidosis, shock	Pale or necrotic mucosa, ulceration	No ischemic changes or abdominal pain present	Feuerstein and Cheifetz [11]
Strongyloidiasis	Abdominal pain, diarrhea, malabsorption	Larvae within mucosa, ulceration	Parasites were luminal and surface‐attached, not intramucosal	Hotez et al. [1]
Schistosomiasis	Portal hypertension, colitis, hematuria	Granulomas, mucosal nodules, eggs	No eggs, granulomas, or vascular changes observed	Hotez et al. [1]

Management priorities focused on stabilizing acute blood loss and eradicating the underlying parasitic infection (Table 3). Given the severity of anemia and ongoing bleeding, red blood cell transfusion was administered to restore oxygen‐carrying capacity and prevent hemodynamic decompensation, consistent with established transfusion thresholds for acute gastrointestinal hemorrhage [12]. Iron‐deficiency anemia was addressed with iron supplementation after bleeding control to replenish depleted stores and support erythropoiesis [13, 15]. Antiparasitic therapy was initiated as definitive treatment, with albendazole selected as first‐line therapy for *Ancylostoma duodenale* and *Necator americanus* , based on its high efficacy and favorable safety profile [14]. Albendazole was administered at a dose of 400 mg orally once daily for three consecutive days, consistent with World Health Organization–recommended regimens for the treatment of hookworm infection [13]. This regimen was selected to ensure effective reduction of worm burden and rapid cessation of mucosal blood loss. A short‐course regimen such as this has been shown to achieve cure rates exceeding 80%–90% and to rapidly reduce worm burden, terminate mucosal feeding, and halt ongoing blood loss [14, 16]. Supportive measures included nutritional optimization, particularly protein and micronutrient intake, to promote mucosal healing and hematologic recovery, and unnecessary acid‐suppressive therapy was discontinued once a non‐acid‐mediated bleeding source had been confirmed [17].

**TABLE 3:** Guideline‐based management of hookworm‐induced acute intestinal bleeding.

Clinical domain	Guideline‐based recommendation	Application in this case	Supporting reference
Initial stabilization	Assess hemodynamic status and correct hypovolemia and anemia in patients with acute gastrointestinal bleeding	The patient was evaluated for hemodynamic compromise and laboratory evidence of severe anemia during active bleeding	Villanueva et al. [12]
Blood transfusion	Transfuse packed red blood cells when hemoglobin < 7 g/dL or in symptomatic anemia to prevent tissue hypoxia	Red blood cell transfusion was administered due to hemoglobin < 7 g/dL with ongoing melena	Villanueva et al. [12]
Identification of bleeding source	Perform upper and lower endoscopy followed by capsule endoscopy when conventional evaluations are nondiagnostic	EGD and colonoscopy were nondiagnostic, prompting capsule endoscopy that identified active hookworm bleeding	Gerson et al. [9]
Antiparasitic therapy	Albendazole or mebendazole is recommended as first‐line therapy for *Ancylostoma duodenale* and *Necator americanus*	Albendazole was administered as definitive therapy for hookworm eradication	WHO [13]; Keiser and Utzinger [14]
Treatment duration	Short‐course albendazole (1–3 days) is effective for moderate‐to‐heavy infections	A short‐course albendazole regimen was used to rapidly reduce worm burden	WHO [13]
Iron replacement	Iron supplementation should follow control of bleeding to restore depleted iron stores	Iron supplementation was initiated after stabilization to treat iron‐deficiency anemia	Camaschella [15]
Nutritional support	Protein and micronutrient supplementation is recommended in parasitic anemia to support mucosal and hematologic recovery	Nutritional optimization was provided to support intestinal healing and erythropoiesis	Camaschella [15]
Acid‐suppressive therapy	Discontinue unnecessary proton‐pump inhibitors when bleeding is not acid‐mediated	Acid‐suppressive therapy was stopped after parasitic bleeding was confirmed	Barkun et al. [16]
Confirmation of eradication	Stool examination using concentration techniques 2–4 weeks after therapy to confirm cure	Follow‐up stool microscopy was planned to document clearance of ova	WHO [13]
Hematologic monitoring	Serial hemoglobin and iron studies to monitor recovery from iron‐deficiency anemia	Hemoglobin and iron indices were followed during recovery	Camaschella [15]
Prevention of reinfection	Counseling on footwear use, sanitation, clean water, and periodic deworming in endemic regions	Preventive counseling was provided due to continued environmental exposure	Pullan et al. [17]
Long‐term surveillance	Monitor for recurrent bleeding, persistent anemia, and malnutrition in heavily infected patients	Ongoing follow‐up was arranged to detect delayed complications	Bethony et al. [2]

The pulmonary emboli incidentally detected on CT angiography required careful therapeutic balancing because of the concurrent active gastrointestinal bleeding. Anticoagulation therapy was therefore initially deferred during the acute bleeding phase, with close cardiopulmonary monitoring while priority was given to stabilization of the hemorrhage and correction of severe anemia [16]. After clinical stabilization and control of gastrointestinal bleeding, anticoagulation therapy was initiated to treat the pulmonary embolism in accordance with standard venous thromboembolism management principles [18]. No additional thrombotic risk factors, including recent immobilization, malignancy, prior thromboembolism, or inherited thrombophilia, were identified during clinical evaluation.

**FIGURE 6:**
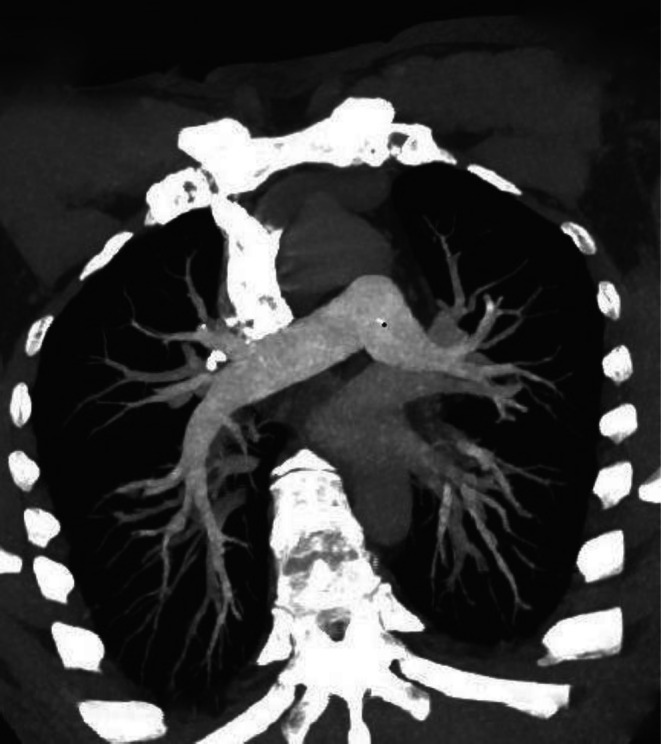
Follow‐up contrast‐enhanced CT pulmonary angiography demonstrating complete resolution of previously detected pulmonary emboli after treatment and clinical recovery.

Follow‐up focused on confirming eradication and monitoring hematologic recovery, with stool examination using concentration techniques planned approximately 2–4 weeks after completion of antiparasitic therapy to document clearance of ova and detect persistent or recrudescent infection [14]. Serial hemoglobin and iron indices were monitored during follow‐up visits over the subsequent weeks after treatment to ensure recovery from iron‐deficiency anemia and to guide the duration of iron supplementation, recognizing that anemia may persist for weeks after parasitic clearance [13]. Follow‐up CT angiography at 6 months demonstrated complete resolution of the pulmonary emboli (Figure 6), confirming recovery from the thromboembolic complication. Given the patient's residence in a hookworm‐endemic region, counseling on footwear use, sanitation, access to clean water, and periodic deworming was provided to reduce the risk of reinfection [18]. Ongoing follow‐up was arranged to monitor for delayed complications such as persistent iron deficiency, malnutrition, or recurrent bleeding, particularly in the context of continued environmental exposure [2].

## 4 Discussion

Direct in vivo visualization of parasite–mucosa interaction in this patient provides compelling evidence that heavy hookworm infestation can produce acute, multifocal small‐bowel hemorrhage through repeated mechanical injury and anticoagulant‐mediated bleeding. Although hookworm‐associated anemia is traditionally attributed to low‐grade daily blood loss, accumulating evidence indicates that high parasite burden and repeated mucosal attachment may generate focal erosions capable of sustaining overt bleeding, particularly when numerous worms feed simultaneously [1, 3]. Human hookworm infections are most commonly caused by *Necator americanus* and *Ancylostoma duodenale* [2]. In sub‐Saharan Africa, including the patient's geographic region, *Necator americanus* is the predominant species, whereas *Ancylostoma duodenale* is more frequently reported in parts of Asia and the Mediterranean [2]. Because species‐level identification was not possible in this case and endoscopic visualization cannot reliably distinguish between hookworm species, regional epidemiology suggests that *Necator americanus* is the most likely causative organism. Capsule endoscopy uniquely enabled dynamic, real‐time visualization of this process, capturing parasite detachment, reattachment, and active hemorrhage—features rarely demonstrable with conventional diagnostic modalities.

Standard esophagogastroduodenoscopy and colonoscopy remain the first‐line investigations for overt gastrointestinal bleeding, but most of the small bowel remains beyond their reach [5, 9]. In endemic regions, this diagnostic blind spot can delay recognition of helminth‐related hemorrhage, leading to repeated negative procedures and prolonged anemia. Multicenter capsule endoscopy series have shown diagnostic yields exceeding 60% for SSBB, with parasitic etiologies accounting for a meaningful proportion of findings in tropical settings [6, 7]. In a study from India, hookworm was identified in nearly 15% of patients with SSBB undergoing capsule endoscopy, emphasizing that this etiology is not rare when specifically sought [7]. The present case reinforces that direct visualization of live parasites attached to bleeding mucosa offers a level of etiologic certainty that cannot be achieved with stool microscopy alone, which often lacks sensitivity in acute bleeding states due to intermittent egg shedding.

The pathobiology observed in this patient aligns with experimental and clinical data showing that hookworms secrete anticoagulant peptides and proteolytic enzymes that prevent clot formation at attachment sites, enabling prolonged blood feeding even after detachment [4]. Repeated migration compounds tissue injury, creating multiple actively bleeding microlesions rather than a single dominant ulcer. This mechanism explains why patients with heavy infestations can develop brisk bleeding disproportionate to the number of worms present and why anemia may progress rapidly despite intact mucosa between attachment sites. Capsule endoscopy uniquely captures this dynamic behavior, distinguishing parasitic hemorrhage from vascular malformations, neoplasia, or inflammatory ulceration.

The detection of pulmonary embolism in this patient represents an important concurrent clinical finding in the setting of severe gastrointestinal bleeding. Although a direct causal relationship cannot be established in this case, severe acute hemorrhage and profound anemia have been associated with physiologic changes such as hemoconcentration, endothelial activation, inflammation, and immobility, which may increase the risk of venous thromboembolism in vulnerable patients [12]. Follow‐up imaging demonstrated resolution of the pulmonary emboli after supportive management; however, any causal relationship between the thromboembolic event and the gastrointestinal bleeding remains uncertain.

Management of hookworm‐induced hemorrhage requires simultaneous stabilization of blood loss and elimination of the parasite. Restrictive transfusion strategies have been shown to improve outcomes in acute gastrointestinal bleeding, but patients with hemoglobin levels below 7 g/dL and ongoing symptoms require red cell transfusion to maintain tissue oxygenation [12, 16]. Iron repletion is equally important, as parasitic blood loss depletes iron stores and limits erythropoietic recovery even after bleeding stops [15]. Anthelmintic therapy with albendazole or mebendazole remains the cornerstone of definitive treatment, with cure rates exceeding 80%–90% in moderate‐to‐heavy infections and rapid reduction in egg output and worm burden [13, 14]. By halting mucosal feeding, these agents directly terminate ongoing hemorrhage and allow mucosal healing.

Beyond individual patient care, this case underscores the importance of integrating environmental and public‐health considerations into the management of parasitic bleeding. Soil‐transmitted helminth infections remain highly prevalent in regions with inadequate sanitation and barefoot agricultural labor, and reinfection after treatment is common without preventive measures [2, 17]. Periodic deworming, footwear use, and access to clean water are therefore essential components of long‐term disease control and anemia prevention, particularly in populations exposed to continual environmental contamination.

From a diagnostic standpoint, this case supports earlier incorporation of capsule endoscopy into the evaluation of SSBB in endemic areas, rather than reserving it as a last resort after multiple nondiagnostic procedures. Current guidelines recognize capsule endoscopy as the preferred first‐line investigation for small‐bowel bleeding once upper and lower endoscopy are unrevealing [9], but its specific diagnostic utility in parasitic disease is often underappreciated. In settings where helminth infections are prevalent, early capsule imaging can expedite diagnosis, avoid unnecessary invasive testing, and allow timely initiation of targeted therapy. Overall, hookworm infestation may present as active small‐bowel hemorrhage, and capsule endoscopy enables definitive identification of this otherwise elusive cause of SSBB in endemic regions.

## 5 Concluding Remarks

Hookworm infestation can cause clinically significant small‐bowel hemorrhage when parasite burden is high, producing multifocal mucosal injury and active bleeding rather than only chronic occult blood loss. Capsule endoscopy enables direct visualization of parasite attachment, migration, and hemorrhage, providing definitive etiologic diagnosis when conventional endoscopy is nondiagnostic. In endemic regions, parasitic infection should be routinely considered in patients with SSBB to allow timely, targeted therapy and prevent ongoing morbidity.

## Author Contributions

**Chukwuka Elendu:** conceptualization, investigation, project administration, supervision, validation, visualization, writing – original draft. **Eunice K. Omeludike:** data curation, methodology, validation, writing – review and editing. **Victor B. Bieh:** formal analysis, methodology, writing – review and editing. **Sobechukwu F. Chiegboka:** data curation, resources, writing – review and editing. **Olusegun A. Aladesanmi:** methodology, visualization, writing – review and editing. **Ebubechukwu Ibe:** data curation, investigation, writing – review and editing. **Ezinnebuchi P. Uzor‐Lawrence:** data curation, resources, writing – review and editing. **Emeka E. Ukoha:** formal analysis, validation, visualization, writing – review and editing.

## Funding

The authors have nothing to report.

## Disclosure

The views expressed in this report are solely those of the author(s) and do not represent the official positions of any affiliated institutions.

## Ethics Statement

Ethical approval was not required, in accordance with the policies of the affiliated institution.

## Consent

Written informed consent was obtained from the patient for publication of this case report and accompanying clinical data.

## Conflicts of Interest

The authors declare no conflicts of interest.

## Data Availability

No datasets were generated or analyzed during the current study, and all relevant clinical information is included within the article.
